# Effects of laboratory colonization on *Bactrocera dorsalis* (Diptera, Tephritidae) mating behaviour: ‘what a difference a year makes’

**DOI:** 10.3897/zookeys.540.9770

**Published:** 2015-11-26

**Authors:** Mark K. Schutze, Thilak Dammalage, Andrew Jessup, Marc J.B. Vreysen, Viwat Wornoayporn, Anthony R. Clarke

**Affiliations:** 1 School of Earth, Environmental and Biological Sciences, Queensland University of Technology, Brisbane 4001 Queensland, Australia; 2 Insect Pest Control Laboratory, Joint FAO/IAEA Division of Nuclear Techniques in Food and Agriculture, International Atomic Energy Agency, Vienna, Austria; 3 NSW Department of Primary Industries, Locked Bag 26, Gosford NSW 2250, Australia

**Keywords:** Oriental fruit fly, sexual compatibility, isolation indices, laboratory adaptation

## Abstract

Laboratory-reared insects are widely known to have significantly reduced genetic diversity in comparison to wild populations; however, subtle behavioural changes between laboratory-adapted and wild or ‘wildish’ (i.e., within one or very few generations of field collected material) populations are less well understood. Quantifying alterations in behaviour, particularly sexual, in laboratory-adapted insects is important for mass-reared insects for use in pest management strategies, especially those that have a sterile insect technique component. We report subtle changes in sexual behaviour between ‘wildish’ *Bactrocera dorsalis* flies (F1 and F2) from central and southern Thailand and the same colonies 12 months later when at six generations from wild. Mating compatibility tests were undertaken under standardised semi-natural conditions, with number of homo/heterotypic couples and mating location in field cages analysed via compatibility indices. Central and southern populations of *Bactrocera dorsalis* displayed positive assortative mating in the 2010 trials but mated randomly in the 2011 trials. ‘Wildish’ southern Thailand males mated significantly earlier than central Thailand males in 2010; this difference was considerably reduced in 2011, yet homotypic couples from southern Thailand still formed significantly earlier than all other couple combinations. There was no significant difference in couple location in 2010; however, couple location significantly differed among pair types in 2011 with those involving southern Thailand females occurring significantly more often on the tree relative to those with central Thailand females. Relative participation also changed with time, with more southern Thailand females forming couples relative to central Thailand females in 2010; this difference was considerably decreased by 2011. These results reveal how subtle changes in sexual behaviour, as driven by laboratory rearing conditions, may significantly influence mating behaviour between laboratory-adapted and recently colonised tephritid fruit flies over a relatively short period of time.

## Introduction

While now debated as to whether it is a driver of speciation, or a secondary effect flowing from population divergence (de Queiroz 1998), the ability to mate and produce viable offspring (sensu the Biological Species Concept (Mayr 1957)) remains, to many biologists, the central test of whether two populations belong to the same or different species (The Marie Curie Speciation Network 2012). Experiments evaluating mating compatibility come with caveats, especially as bringing two populations together under artificial circumstances may influence one or more critical steps of the mate recognition process. Only when random mating between known conspecifics is demonstrated in choice mating tests, and incompatibility with a second putative species, then strong inference as to species limits can be drawn (Walter 2003).

For tephritid fruit flies, cross-species mating in small cages results in forced matings that produce viable hybrids (Cruickshank et al. 2001), and so are inappropriate for use in delimiting species boundaries. In contrast, mate choice experiments in large, walk-in field cages containing a host plant (i.e., semi-natural conditions) have proven useful tools in discriminating among closely related sibling species (Petit-Marty et al. 2004, Vera et al. 2006, Cáceres et al. 2009, Schutze et al. 2013, Bo et al. 2014). As reviewed by Juárez et al. (2015), protocols for such trials are now well established and widely applied. Where it is logistically feasible to bring populations together, such mating tests are a recommended component of integrative taxonomic studies (sensu Schlick-Steiner et al. 2010) on frugivorous tephritids (Clarke and Schutze 2014).

International protocols for tephritid mating trials were initially designed to test competiveness and compatibility among flies mass-reared for the sterile insect technique (SIT) and their wild counter-parts, or to compare the competitiveness and compatibility of populations from different mass-rearing facilities in different geographical areas (FAO/IAEA/USDA 2003). As such, they were factory quality assurance tests that were developed with a need for clearly defined, easily repeatable, and statistically comparable data sets. The key parameters now commonly used for tephritid mating trials (the Index of Sexual Incompatibility (ISI) and the Male and Female Relative Performance Indices (MRPI and FRPI, respectively)) meet these needs, but when used alone may hide potentially critical biological information. Most importantly, these indices report who eventually mated with whom, but not why, or more tellingly, why they did not. While the collection and reporting of secondary behavioural data (e.g., mating time and location in the field cage) in tephritid mating studies is recommended in the international protocol, its importance appears often downplayed in the literature when compared to the reporting of the main mating indices.

In this paper, we report on two crossing experiments using the same populations of *Bactrocera dorsalis* (Hendel), conducted under identical experimental conditions exactly 12 months apart. The first cross used nearly wild flies (F1-F2 generation), while the second cross used flies from the same colony when six generations in culture (F6). Mating compatibility between the two populations was assessed between the trials, and this example was used to discuss: i) the importance of collecting secondary behavioural data in mating trials; ii) the importance of understanding subtle differences in courtship behaviour which may occur between wild populations of the same species; and iii) the implications of using ‘wildish’ versus laboratory-adapted populations for integrative taxonomic research.

## Methods

### Source material

We evaluated mating behaviour of *Bactrocera dorsalis* from central and southern Thailand. All flies were sourced directly from the wild via host-rearing and sent to the FAO-IAEA Insect Pest Control Laboratory (IPCL), Seibersdorf Austria, in March 2010. Collection locations were not privately owned and no endangered or protected species were involved in the study. No specific permits were required for the described field studies or for the import of live material into the IPCL. The central Thailand population was reared from *Mangifera indica* L. (Anacardiaceae) in Saraburi and sent as a batch of approximately 500 pupae; the southern population was reared from *Carica papaya* L. (Caricaceae) in Nakhon Si Thammarat and sent as a batch of approximately 200 pupae.

Flies were morphologically examined for external and internal genitalic characters to confirm their identity in accordance with taxonomic descriptions (Drew and Hancock 1994). Professor R.A.I. Drew confirmed the identity of the cultures as *Bactrocera dorsalis* based on pinned material, diagnostic micrographs, and genitalia measurements. Further, material from these colonies were used in subsequent integrative taxonomic studies examining molecular and morphological characters that further confirmed both colonies as *Bactrocera dorsalis* (Krosch et al. 2013, Boykin et al. 2014). Representative voucher samples were preserved as dried (pinned) and wet (> 95% alcohol) material at the IPCL and Queensland University of Technology, Brisbane, Australia.

### General rearing protocol

Adult flies were provided a standard diet of enzymatic yeast hydrolysate and sugar (1:3) together with water *ad libitum*. Sexually mature flies were exposed to egg-cups dosed with commercial guava juice (Rubricon, Rubricon Products, Middlesex, U.K.) as an oviposition stimulant. Eggs were incubated overnight (25 ± 2 °C, 65% R.H.) on moist filter paper placed on wet sponges in Petri dishes and then transferred to carrot diet (Tanaka et al. 1970) for larval development (27–28 °C, 55% R.H.). Pupae were collected into and sifted from moistened teak sawdust, and transferred to either experimental (20 cm diameter × 27 cm height) or colony cages (50 cm × 50 cm × 50 cm).

### Mating compatibility tests

The first mating compatibility tests were conducted in June and July of 2010 when Saraburi and Nakhon Si Thammarat colonies were at the F1/F2 laboratory colony generation. Eight replicates were completed, consisting of five using F1 generation flies and three using F2 generation flies. The second series of mating compatibility tests were undertaken one-year later in July 2011 when both cultures had reached F6 (eight replicates completed). Experimental protocols were identical for 2010 and 2011 trials, as outlined below.

Flies were sexed within four days of emergence; this is well before male and female sexual maturation which occurs 15-20 days post emergence based on personal observation (MKS; data not shown) and previous studies (McInnis et al. 1999, Wee and Tan 2000). Flies were maintained under low-stress conditions of 100-200 flies per cylindrical cage (20 cm diameter × 27 cm height). General procedures followed the FAO/IAEA/USDA (2003) Manual for Product and Quality Control. A small dot of coloured water-based paint was applied to the dorsal surface of each fly’s thorax using a soft paint-brush (colours were randomized among tests) for identification of each population. Painting was done at least 48 hours prior to each field cage test to allow paint to dry and flies to become habituated to its presence. Mating tests were undertaken using flies between 20-30 days of age to ensure sexual maturity had been reached by the majority of individuals (> 90% of wild *Bactrocera dorsalis* reach maturity by 24 days (Wong et al. 1989)).

Field cage tests were conducted inside a glasshouse exposed to natural light and maintained at ~25 °C and ~50% R.H. Replicates were undertaken inside one of four partitioned flight cages (2.0 m × 1.6 m × 1.9 m) within the glasshouse, with each cage containing a single, non-fruiting potted *Citrus sinensis* Osbeck (Rutaceae) tree of 2 m in height with a canopy of ~ 1.1 m in diameter.

Flies were released into the experimental field cage at a 1:2 male:female ratio. As this study was focussed on mating compatibility and not strictly competition, this ratio of males to females (as opposed to 1:1) was used to ameliorate the effect of potentially early-mating males from monopolising all females from one population and thus inflating isolation indices, as per Schutze et al. (2013). By providing twice as many females as males, potentially later starting males still have access to females from both populations. As *Bactrocera dorsalis* mates at dusk (Arakaki et al. 1984), 20 males of each of the two populations were released into a field cage one to two hours prior to sunset for each replicate; 40 females of each of the same two populations were released 30 min later for a total of 40 males and 80 females per replicate. Experimental observations began immediately after females were released. Once couples formed, they were gently coaxed into sequentially numbered plastic vials (3.7 cm diameter × 4.0 cm). The following data were recorded for each pair: male origin; female origin; time of mating; and position (cage or tree). Periodic measurements of temperature (°C) and relative humidity in the cage were also made. Experiments concluded when flies became inactive, which occurred after sunset when light intensity dropped below 10 lux.

### Data analysis

Relative percentages of each of the four possible couples (i.e., Saraburi ♂ × Saraburi ♀ [SS], Saraburi ♂ × Nakhon Si Thammarat ♀ [SN], Nakhon Si Thammarat ♂ × Saraburi ♀ [NS], and Nakhon Si Thammarat ♂ × Nakhon Si Thammarat ♀ [NN]) were calculated for each replicate. Proportion data were arcsine transformed prior to subsequent analysis; one-way anova (with Tukey *post hoc* test where appropriate) was conducted to assess for significant differences among mating combinations within each year; paired t-tests were conducted to assess for significant differences in relative proportions of respective couple combinations across years.

Compatibility was determined using the Index of Sexual Isolation in conjunction with the Male Relative Performance Index and the Female Relative Performance Index (Cayol et al. 1999). Values of ISI may range from +1 (complete positive assortative mating, i.e., males and females only mating with their respective population) to 0 (complete random mating) to -1 (complete negative assortative mating; i.e., all males of one population mating with all females of the opposite population and *vice versa*). Values of MRPI range from +1 (only males of one population mated; i.e., the first listed in the test) to 0 (males of both populations participated equally in mating) to -1 (only males of the reciprocal population mated; i.e., the second listed in the test). The FRPI is the equivalent of the MRPI but as applied to females.

Ninety-five percent confidence intervals of the used indices (ISI, MRPI, and FRPI) for each of 2010 (F1/F2 flies) and 2011 (F6 flies) were calculated to determine deviations from random mating (ISI = 0) or equal participation by the respective sexes (MRPI & FRPI = 0). Confidence intervals that included zero represent cases of random mating and equal participation between the populations. Heterogeneity chi-square analyses across replicates for each treatment were undertaken to determine if data could be combined prior to further analysis. Following heterogeneity tests, chi-squared tests of independence were applied to determine if males mated predominantly with females of one population over the other.

The mean time to begin mating (mating latency) was estimated by calculating how many minutes had elapsed between the time each couple initiated mating and the time of the first observed mating couple (= time zero) within each particular cage replicate (as per Schutze et al. 2013). Statistical analyses were conducted for each of the tests, with latency data for each of the four possible mating combinations combined prior to one-way ANOVA (with Tukey *post hoc* test where appropriate) to determine significant differences (α = 0.05) in latency among mating combinations. We conducted a one-way ANOVA (with Tukey *post hoc* test where appropriate) on arcsine transformed percentage data of couples collected on the tree for each trial to determine if there was a significant difference in couple location (cage or tree).

All values reported represent mean ± 1 s.e. unless otherwise stated.

## Results

Eight replicates of mating compatibility tests were completed for each of the 2010 and 2011 trials; 84.7 ± 2.1% and 78.1 ± 4.3% flies mated in 2010 and 2011, respectively.

**Figure 1.**
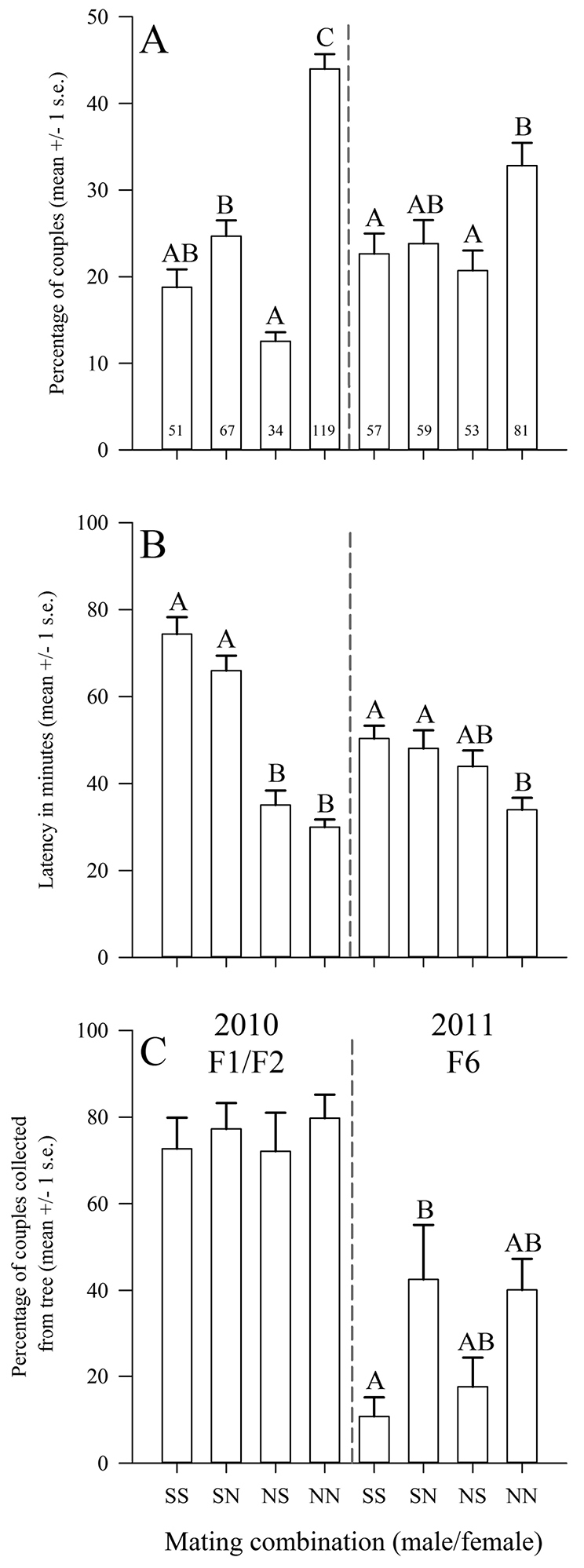
Behavioural parameters of *Bactrocera dorsalis* flies from Saraburi (S) and Nakhon Si Thammarat (N) (Thailand) during mating compatibility trials in 2010 and 2011. **A** Relative percentages and total numbers of each possible couple formed. Numbers in bars are total numbers of each couple formed summed across replicates **B** Mating latency as average time since first couple observed for couples formed **C** Average percentage of respective couples collected from the tree for each of the six mating compatibility comparisons. For all graphs, columns surmounted by the same letter within a year are not significantly different at α = 0.05.

Total numbers and mean percentages of each of the four possible mating-pair combinations (i.e., SS, SN, NS, and NN) varied considerably between years (Figure 1A). There was a highly significant difference among the percentages of mating combinations in 2010 (F_(3,28)_ = 56.59, *p* < 0.0001), with significantly more NN couples (43.99 ± 1.70%) forming relative to all other couples, together with significantly more SN heterotypic couples forming relative to the reciprocal NS. There was a significant difference among the percentages of mating combinations in 2011 (F_(3,28)_ = 4.44, *p* < 0.05), with significantly more NN couples (32.82 ± 2.62%) forming relative to SS and NS couples. While in 2010 there was an increased difference in the numbers of couples other than NN (i.e., SS, SN, and NS), there was no such difference among these couple combinations in 2011, with heterotypic couples (SN and NS) equally represented as SS homotypic couples; indeed, numbers of SN couples was not statistically different to NN couples. Across years, there was a significant change in relative proportion of NN and NS couples from 2010 to 2011 (*t* = 3.539, df = 14, *p* < 0.01; *t* = -3.410, df = 14, *p* < 0.01, respectively); however, there was no significant change in the relative proportion of SN and SS couples across years (*t* = 0.326, df = 14, *p* = 0.749; *t* = -1.250, df = 14, *p* = 0.232, respectively).

Analysis of latency (time to mate since first couple formed) revealed further differences between populations of *Bactrocera dorsalis* from Nakhon Si Thammarat and Saraburi (Figure 1B). There was a significant difference in latency between couples involving males from Saraburi and males from Nakhon Si Thammarat in 2010 (F_(3,266)_ = 61.18, *p* < 0.0001). Couples with males from Saraburi mated significantly later (SS = 74.34 ± 3.93 min and SN = 65.99 ± 3.46 min) than those from Nakhon Si Thammarat (NS = 35.03 ± 3.32 min and NN = 29.94 ± 1.73 min). While this trend continued in 2011 with significant differences in latency among couple combinations (F_(3,246)_ = 5.562, *p* < 0.05), the difference was nevertheless reduced with males from Saraburi mating sooner after sunset compared with those involved in the F1/F2-generation 2010 trials (2011 latency for SS = 50.35 ± 2.96 min; SN = 48.10 ± 4.17 min) while latency of couples involving males from Nakhon Si Thammarat either increased slightly or remained approximately the same (NS = 43.96 ± 3.65 min; NN = 33.95 ± 2.74 min). Overall, there were no significant differences in latency among SS, SN, or NS couples in 2011; however, NN couples still mated significantly sooner than SS and SN (i.e., those involving Saraburi males) as for the 2010 trial.

There were no significant differences among mating combinations with respect to position on the tree or cage wall for the 2010 trial (F_(3,28)_ = 0.134, *p* = 0.939); however, there was a significant difference among couples in the 2011 trial (F_(3,28)_ = 3.902, *p* < 0.05) (Figure 1C). In 2011, SS couples mated significantly more on the cage wall (10.76 ± 4.37% on the tree) compared to SN couples of which 42.52 ± 12.58% of couples mated on the tree. While statistically non-significant, other combinations displayed similar trends, with only 17.59 ± 6.75% of NS couples mating on the tree in contrast to 40.11 ± 7.16% of NN couples on the tree. Taken together, and calculated based on summed replicates, 2011 couples that involved females from Nakhon Si Thammarat mated on trees 37% of the time relative to 15% of couples involving females from Saraburi.

**Figure 2.**
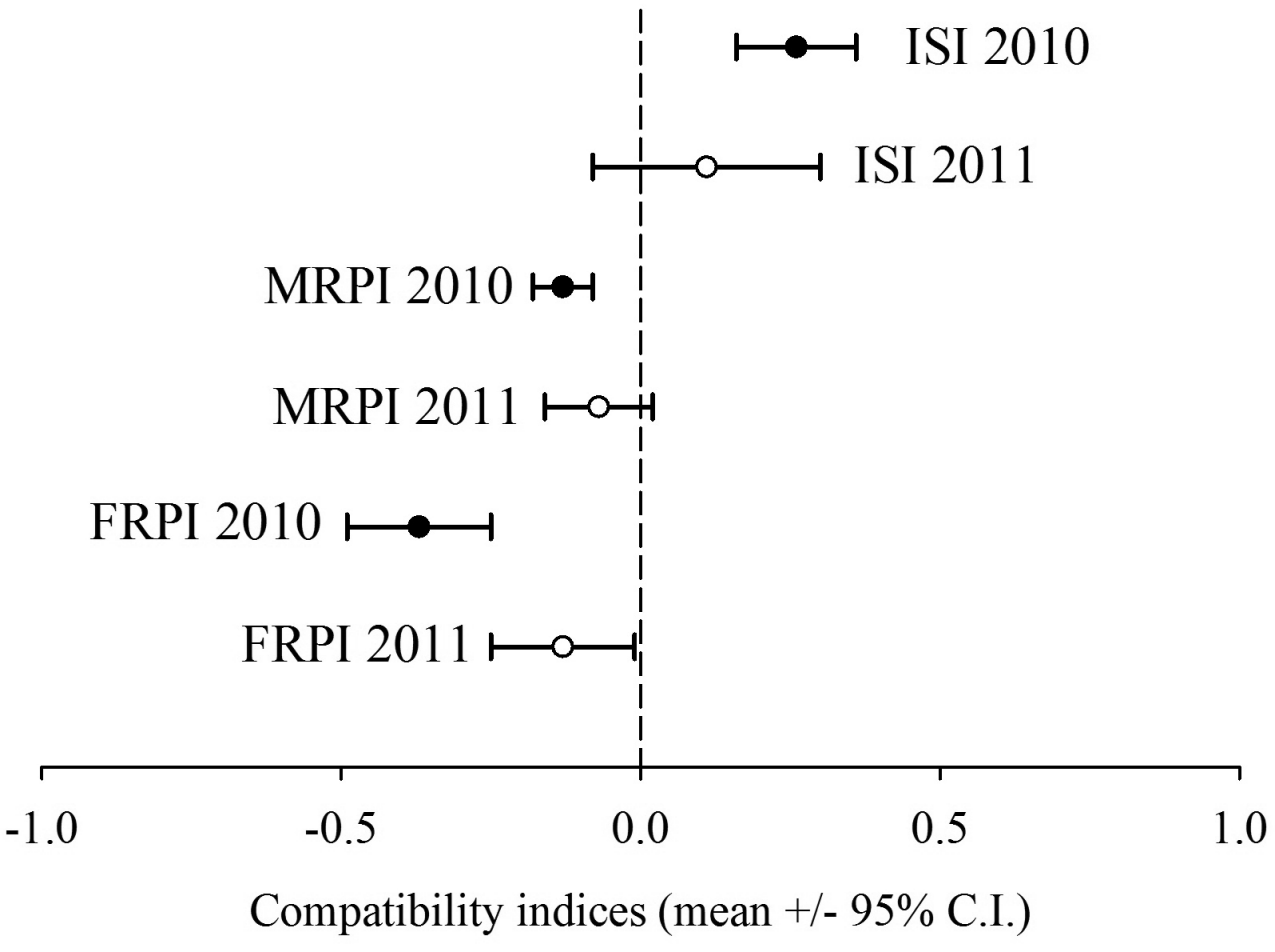
Index of Sexual Isolation (ISI) and relative performance indices for males (MRPI) and females (FRPI) with associated 95% confidence intervals calculated for 2010 and 2011 mating compatibility comparisons between *Bactrocera dorsalis* from Saraburi and Nakhon Si Thammarat, Thailand. Dotted line (0.00) represents random mating (ISI) or equal participation by the sexes (MRPI and FRPI).

As chi-squared tests of independence were homogeneous across replicates for both years (2010 χ^2 ^= 3.49, df = 7, *p* = 0.836; 2011 χ^2 ^= 11.18, df = 7, *p* = 0.131), data were summed prior to analysis of mating indices ISI, MRPI and FRPI. While there was a significant bias towards assortative mating in 2010 (χ^2 ^= 13.64, df = 1, *p* < 0.0001; ISI = 0.26 ± 0.19 [95% C.I.]), this effect was largely lost by the time F6 flies were crossed in 2011, despite the consistent and significant increase in number of NN couples (χ^2 ^= 2.32, df = 1, *p* = 0.128; ISI = 0.11 ± 0.10 [95% C.I.]) (Figure 2).

The FRPI significantly deviated from random (FRPI = -0.37 ± 0.12 [95% C.I.] in 2010, reinforcing that considerably more Nakhon Si Thammarat females mated (n = 186 summed across reps for SN and NN) relative to those from Saraburi (n = 85 summed across reps for SS and NS). While this trend continued in 2011, there was a considerably reduced difference in female participation (n = 140 *versus* n = 110, resp.) as reflected in the FRPI measure approaching zero (FRPI = -0.13 ± 0.12 [95% C.I.]). While less dramatic, there were also significantly more males from Nakhon Si Thammarat mating (n = 153 summed across reps for NS and NN) relative to those from Saraburi (n = 118 summed across reps for SS and SN) in 2010 with a mean MRPI (± 95% C.I.) of -0.13 ± 0.05 (Figure 2); yet in 2011 this difference in relative male participation was, as for females, considerably reduced with a total of 134 Nakhon Si Thammarat males mating (NS and NN summed across reps) compared to 116 from Saraburi (SS and SN summed across replicates).

## Discussion

### Changes in mating behaviour over a year

Our results show that F1/F2 (= ‘wildish’) *Bactrocera dorsalis* from Saraburi and Nakhon Si Thammarat demonstrated significant positive assortative mating: i.e., like was more likely to mate with like than expected by chance. This assortative mating was lost by the 6^th ^generation, when random mating occurred between the two populations. The change from positive assortative to random mating was most likely due to two factors: latency and relative participation of the sexes. In ‘wildish’ populations Nakhon Si Thammarat males mated sooner than Saraburi males (i.e., their mating latency time was shorter) and Nakhon Si Thammarat females mated more than Saraburi females. The combination of the two attributes led to more Nakhon Si Thammarat × Nakhon Si Thammarat matings. By the 6^th ^generation, the temporal difference in male latency was lost, as was the increased ‘precociousness’ of the Nakhon Si Thammarat females, leading to random mating between the populations.

Differences in latency in male mating behaviour may be the results of local environmental conditions from where respective populations of *Bactrocera dorsalis* originated. Time of sunset, for example, may be a potential causal factor, considering Nakhon Si Thammarat is located approximately 600 km south of Bangkok and time of sunset (and time of mating) would correspondingly vary. However, despite their geographic distance, time of sunset differs little between these locations across the year: the sun sets approximately 10-12 minutes later in Nakhon Si Thammarat relative to Bangkok in January, yet in July it sets approximately 6 minutes earlier (based on 2014 sunset data; www.sunrise-and-sunset.com). Nevertheless, this slight difference may be sufficient to influence mating latency in early-generation laboratory colony flies. Complexity in circadian rhythm patterns and differences in mating latency between wild and mass-reared colonies have been investigated in other tephritid species, such as the melon fly *Bactrocera cucurbitae* (Coquillett) (Matsuyama and Kuba 2009); exemplifying how subtle, but significant, differences in the onset of mating behaviour can be readily manipulated by changes in daily light patterns (Miyatake et al. 2002). We cannot rule out other factors besides time of sunset, however, and further work into the driving mechanisms of *Bactrocera dorsalis* mating latency are warranted.

What influenced variation in female mating in our trials remains open to speculation. Drivers of sexual propensity are varied, and may include both intrinsic and extrinsic (e.g., temperature, food, density, and sex ratios) factors (Spiess 1970). Differences in mating propensity may also be affected by body size, as demonstrated in other tephritids such as *Ceratitis capitata*, for which mating frequency was dependant on the relative sizes of males and females (Churchill-Stanland et al. 1986). We did not, however, record additional attributes (e.g., size) of flies used in these trials; therefore, we are unable to account for the contribution of any of these factors towards differences in mating propensity of either sex.

### ‘Wildish’ or laboratory adapted flies

These results pose a conundrum for mating trials. It is generally considered that the use of ‘wildish’ populations (i.e., within one or very few generations of field collected material) is more desirable than using flies that already have been cultured for a long time because laboratory selection may alter key behavioural and physiological traits (Miyatake 1998, 2011, Meats et al. 2004, Gilchrist et al. 2012). However, in our case the use of ‘wildish’ populations led to a result which, when taken alone, was contrary to additional evidence that *Bactrocera dorsalis* constitutes a single biological species within Thailand (Schutze et al. 2012, Krosch et al. 2013, Aketarawong et al. 2014, Boykin et al. 2014). That is, we observed significant assortative mating behaviour driven by behavioural differences which may support a two-species hypothesis; yet, all other forms of data (e.g., molecular, morphological, cytogenetic, and chemoecological) strongly infer con-specificity across Thailand. Nevertheless, subtle differences in mating latency between populations of other *Bactrocera* species are known, even when there is no suggestion of sibling species. For example, differences in mating latency between populations of *Bactrocera cucurbitae* have been recorded (Miyatake 1998, 2011), but there is no evidence for cryptic taxa within that species (Hu et al. 2008, Virgilio et al. 2010).

Based on this experiment alone we are not in a position to make strong statements about using ‘wildish’ versus older cultures for mating tests in *Bactrocera dorsalis*, but we do highlight that even within a single biological species, local adaptation and drift may lead to subtle but potentially important differences in some aspects of the mating system, as documented in other organisms (Schmitt and Gamble 1990, Berlocher and Feder 2002, Nosil et al. 2005, Cocroft et al. 2010). Perhaps a compromise to ameliorate potential short term behavioural ‘hangovers’ from wild populations is to rear populations for a pre-determined number of generations to standardise colonies in the laboratory, so that any differences found are more likely to reflect underlying genetic causes and not short-term environmental influences. That said, long-term rearing does lead to significant laboratory adaptation, and as demonstrated for some species this bottleneck can occur within the first few generations (Frankham and Loebel 1992, Hoffmann et al. 2001, Gilchrist et al. 2012). The significant reduction in the number of matings occurring on foliage versus cage walls from the F1/F2 flies to the F6 flies is perhaps behavioural evidence of laboratory adaptation, and for within *Bactrocera dorsalis* we suggest conducting compatibility studies on colonies greater than two generations from the wild, but not greater than six.

### Mating indices as a factory QA measure versus a research tool

As a factory quality assurance measure, mating indices serve a valuable function by allowing repeatable and quantifiable measures of the quality of factory flies, thereby forming an effective tool allowing SIT action programme managers to determine if sterile males are fit for purpose to compete with wild males and are compatible with wild females. But there is no doubt that these indices, by focusing exclusively on the ‘end product’ of copulation, may lead some researchers to potentially under appreciate biologically important steps in the courtship process. Where mating is 100% random, or 100% positive-assortative, these prior steps may be less critical for interpreting meaning from the trials. But where the results fall in between these extremes, as has been found in several *Bactrocera* and *Anastrepha* studies (Schutze et al. 2013, Vera et al. 2006), the absence of more detailed courtship or mating data makes interpretation difficult.

## Conclusion

The use of very recently established colony material is widely considered ideal for determining mating compatibility among strains, populations, or putative species. Our results clearly demonstrate that subtle behavioural characteristics may ‘carry-over’ from the wild and may result in inflated measures of incompatibility that are soon lost following colony establishment. For future tephritid research where mating is used to help delimit cryptic species, we therefore encourage the use of detailed courtship behaviour in field cage mating studies that is quantified by isolation and additional indices that dissect specific behavioural attributes among populations or putative species.
